# Use of a Pathomics Signature to Predict the Prognosis of Hepatocellular Carcinoma with Cirrhosis: A Multicentre Retrospective Study

**DOI:** 10.3390/cancers17193192

**Published:** 2025-09-30

**Authors:** Ting Wang, Jixiang Zheng, Lingling Guo, Jiawen Fan, Yubin Lu, Zhen Peng, Yanfeng Zhong, Zhengjun Zhou, Erbao Chen

**Affiliations:** 1 Department of Hepatobiliary and Pancreatic Surgery, Peking University Shenzhen Hospital, Shenzhen 518036, China; wangtingofsmu@163.com (T.W.); pz17718153297@outlook.com (Z.P.); 2 Department of General Surgery, Guangzhou Digestive Disease Center, Guangzhou First People’s Hospital, Guangzhou Medical University, Guangzhou 510180, China; zjx15626409167@163.com; 3 Department of Radiation Oncology, Peking University Shenzhen Hospital, Shenzhen 518036, China; guolling3@mail3.sysu.edu.cn; 4 Department of Gastrointestinal Surgery, Shenzhen Hospital of Southern Medical University, Shenzhen 518100, China; fanjiawen17@163.com; 5 Minimally Invasive Interventional Department, Peking University Shenzhen Hospital, Shenzhen 518036, China; 2100251026@email.szu.edu.cn; 6 Central Laboratory, Peking University Shenzhen Hospital, Shenzhen 518036, China; zhongyf9785@163.com; 7 Liver Cancer Institute, Zhongshan Hospital, Fudan University, Shanghai 200032, China

**Keywords:** hepatocellular carcinoma, cirrhosis, pathomics, prognosis prediction

## Abstract

Background: Hepatocellular carcinoma (HCC) is a highly aggressive and heterogeneous malignancy which predominantly arises in the setting of cirrhosis, and there is lack of models to predict prognosis in cirrhotic HCC. This study aims to develop and validate a prediction model based on the pathomics signature and clinicopathological characteristics to predict the prognosis of HCC with cirrhosis. Methods: In this multicenter, retrospective study, 389 patients were enrolled (training cohort: 268; independent validation cohort: 121). A total of 351 pathomics features were extracted from digital H-E–stained images, and a pathomics signature (PS_HCC_) was constructed using a least absolute shrinkage and selection operator Cox regression model. Then two nomograms were established by combining the PS_HCC_ and clinicopathological characteristics. Further validation was performed in the validation cohort. Results: This study included 389 patients. A 24 feature-based PS_HCC_ was constructed. A higher PS_HCC_ was significantly associated with worse OS and DFS in both the training (OS: hazard ratio [HR], 4.341 [95% CI, 3.109–6.062]; DFS: HR, 3.058 [95% CI, 2.223–4.207]) and validation (OS: HR, 4.145 [95% CI, 2.357–7.291]; DFS: HR, 3.395 [95% CI, 2.104–5.479]) cohorts (*p* < 0.001 for all comparisons). Multivariable analysis revealed that the PS_HCC_ was an independent factor associated with OS and DFS. Integrating the PS_HCC_ into pathomics nomograms resulted in better performance for prognosis prediction than the traditional model in both cohorts. Conclusions: The PS_HCC_ may serve as a reliable surrogate for prognosis, and the nomograms offer promising tools to predict individual outcomes, facilitating personalized management of HCC with cirrhosis.

## Simple Summary

Prognostic models are lacking for cirrhotic hepatocellular carcinoma (HCC). We constructed a pathomics signature based on pathomics features extracted from digital H-E images and LASSO Cox regression, which was easy to reproduce and may be conveniently applied in clinical practice. The pathomics signature is the independent predictor of OS and DFS, and the nomograms incorporating the pathomics signature and clinicopathological factors outperformed the traditional model consisting of clinicopathological factors alone. These findings may contribute to the use of the pathomics signature in clinical practice and facilitate personalized treatment strategies for HCC with cirrhosis.

## 1. Introduction

Hepatocellular carcinoma (HCC), the most prevalent liver malignancy, ranks as the third leading cause of cancer-related mortality globally [1]. While hepatectomy remains a curative option for specific patients with HCC, recurrence occurs in 50–70% of cases within five years, and the overall 5-year survival rate ranges from 25% to 55% [2,3,4,5]. Notably, HCC is distinguished by its association with cirrhosis in 90% of cases, with primary causes including hepatitis B or C virus, nonalcoholic fatty liver disease, and alcohol-related liver disease [6]. Patients with cirrhosis face a heightened risk of postoperative complications and a poorer prognosis than those without [7]. Accurate prognostic assessment in cirrhotic HCC remains a critical challenge for personalized treatment approaches.

Current HCC staging systems, such as the Barcelona Clinic Liver Cancer (BCLC), Hong Kong Liver Cancer (HKLC), and American Joint Committee on Cancer (AJCC) TNM systems, have been employed to perform prognostic stratification and inform treatment decisions in HCC [1,2,3,4]. However, these models fail to account for the immunosuppressive environment induced by cirrhosis, limiting their efficacy in precise risk stratification [8]. Consequently, there is an urgent need for predictive models to optimize individual outcome prediction and treatment strategies, particularly for patients with cirrhosis.

Hematoxylin and eosin (H&E)-stained slides provide critical diagnostic information and contain vast morphometric data that are potentially indicative of survival outcomes—details often beyond the scope of routine pathological evaluation. Advances in digital pathology and artificial intelligence have enabled deeper extraction of these features, giving rise to the field of “pathomics,” which has garnered increasing interest [9,10]. Pathomics encompasses the quantitative extraction of data from digital histopathology images, capturing cellular architecture and histological texture in both tumor cells and the surrounding extracellular matrix [11,12,13]. Unlike traditional staging systems, pathomics offers a comprehensive characterization of the HCC tumor microenvironment. Previous studies have demonstrated its utility in predicting outcomes for lung, bladder, renal, gastric, and colon cancers [14,15,16,17,18]. Building on this, we hypothesized that pathomics analysis of digital H&E-stained images could enhance prognostic predictions for cirrhotic HCC.

Integrating multiple features into a unified signature, as opposed to analyzing them individually, has the potential to significantly enhance predictive accuracy [19,20]. The least absolute shrinkage and selection operator (LASSO) Cox regression, a powerful machine learning algorithm for high-dimensional data, is widely applied in prognostic modeling [21,22,23]. In this study, we employed LASSO Cox regression to derive a pathomics signature for HCC (PS_HCC_) based on quantitative features extracted from digital H&E images. Subsequently, we evaluated the prognostic efficacy of the PS_HCC_. To facilitate clinical application, we established and validated two pathomics nomograms, incorporating both PS_HCC_ and clinicopathological factors, to individually predict overall survival (OS) and disease-free survival (DFS) in cirrhotic patients with HCC.

## 2. Materials and Methods

### 2.1. Study Design

**Figure 1:**
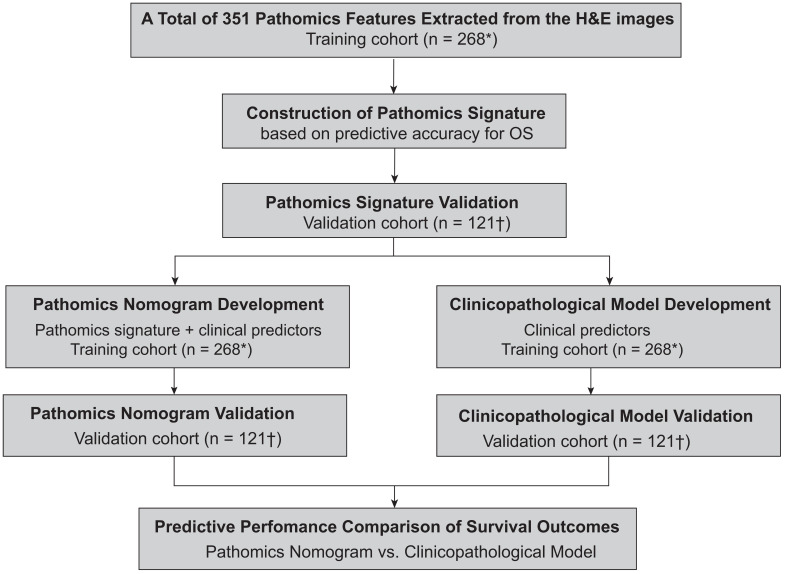
Flowchart of the study. * Patients from Fudan University Zhongshan Hospital. † Patients from Peking University Shenzhen Hospital.

The study design is illustrated in Figure 1. In the training cohort, a total of 351 pathomics features were obtained, followed by the construction of a pathomics signature (PS_HCC_) based on OS-associated features identified via LASSO Cox regression. Two pathomics nomograms, incorporating both the PS_HCC_ and clinical predictors, were then constructed to predict prognosis in HCC with cirrhosis. Their predictive performances were validated in the external validation cohort. Additionally, two clinicopathological models were established to evaluate the incremental prognostic value provided by the PS_HCC_.

### 2.2. Participants

This retrospective study included 389 HCC participants with cirrhosis, sourced from two medical centers. The training cohort comprised 268 consecutive patients from Zhongshan Hospital, Fudan University, received curative resection between 1 July 2010 and 31 March 2013. Inclusion criteria were (1) histologically confirmed HCC, treated with curative resection; (2) Child–Pugh class A or B with liver cirrhosis; (3) no prior history of other malignancies; and (4) availability of comprehensive clinicopathological and follow-up data. Patients who underwent repeat hepatectomy, local ablation, or transcatheter arterial chemoembolization prior to surgery were excluded. The validation cohort, consisting of 121 patients, was selected from Peking University Shenzhen Hospital between 1 July 2008 and 31 March 2011, using identical inclusion and exclusion criteria.

Baseline patient data, including age, sex, HBV infection, Child–Pugh grade, AFP level, tumor number, tumor size, differentiation grade, encapsulation, vascular invasion, TNM stage, and follow-up information, were collected. Follow-up occurred every three months during the first two years post-surgery, every six months for the subsequent three years, and annually thereafter. Follow-up duration was measured from the surgery date to the last follow-up. In this study, curative resection was defined as complete macroscopic removal of tumor, as confirmed by intraoperative inspection and postoperative imaging (CT/MRI within four weeks after surgery) and surgical margins were histologically negative (R0 resection) for cancer cells. This study included HCC patients with cirrhosis who have worse survival than those without cirrhosis [7]. Thus, a high rate of early recurrence is reported despite enrolled patients receiving curative resection.

This study was in accordance with the Declaration of Helsinki and approved by the Institutional Review Boards of Fudan University Zhongshan Hospital (approval number: B2024-199, date: 31 May 2024) and Peking University Shenzhen Hospital (approval number: 2022-164, date: 2 December 2022). Written informed consent was waived by the institutional review boards for this retrospective study. The study followed the TRIPOD (Transparent Reporting of a Multivariable Prediction Model for Individual Prognosis or Diagnosis) statement guidelines.

### 2.3. Sample Preparation and Region of Interest Selection

Initially, two experienced pathologists, blinded to the prognostic data, reviewed the H&E-stained slides and selected the most representative samples. Paraffin-embedded tissue sections were then cut at a thickness of 5 μm and stained with H&E. All slides were subsequently scanned using the Aperio ScanScope system at ×20 magnification, digitized into svs format files. Under the supervision of an experienced pathologist, the tumor regions in each section were identified. To enhance efficiency, a pathologist selected ten non-overlapping representative tiles per core, each containing the most tumor cells, with a field of view of 1000 × 1000 pixels (0.504 μm/pixel). The selected tiles were confirmed by a second pathologist, and disagreements were resolved through consultation with the pathology director. All pathologists remained blinded to clinical and prognostic data. The selected tiles were saved in tif format.

### 2.4. Pathomics Feature Extraction

Pathomics features were extracted from digital H&E-stained images using CellProfiler, an open-source software (version 4.2.1; Broad Institute; Cambridge, MA, USA, https://cellprofiler.org/, accessed on 5 January 2023) [24]. Initially, the selected H&E-stained tiles were split into hematoxylin-stained and eosin-stained grayscale images via the “UnmixColors” module. Additionally, the H&E-stained tiles were converted to grayscale using the “ColorToGray” module with the “Combine” method for subsequent processing. Image quality metrics for the grayscale, hematoxylin, and eosin channels were evaluated through the “MeasureImageQuality”, “MeasureImageIntensity”, and “MeasureObjectIntensity” modules. Pixel-level colocalization and intensity correlation features across an entire image were obtained using the “MeasureColocalization” module. Granularity features, reflecting the spectra of size measurements of textures within the image, were quantified via the “MeasureGranularity” module. Finally, texture features, which describe the roughness and smoothness of textures within the images, were derived using the “MeasureTexture” module. A total of 351 features were obtained and presented in Table S1.

### 2.5. PS_HCC_ Construction

LASSO Cox regression, a robust method for high-dimensional predictor analysis in survival studies, was used [16,17]. It employs an L1 penalty to shrink coefficients toward zero, with the tuning constant λ controlling the penalty strength. A smaller λ allows more predictors into the model. The optimal value of λ is determined by 10-fold cross-validation. In the training cohort, we used LASSO Cox regression to select the most prognostic pathomics features and constructed a formula based on a linear combination of the selected features to calculate the pathomics signature (PS_HCC_). The PS_HCC_ for the validation cohort was calculated using the derived formula. The PS_HCC_ calculation formula is presented in the Supplementary File S1.

### 2.6. Prognostic Value of the PS_HCC_

The optimal PS_HCC_ cutoff value was established using maximally selected rank statistics, dividing patients in the training cohort into high- and low-PS_HCC_ groups. The same cutoff was utilized to stratify patients in the validation cohort. The Kaplan–Meier method and log-rank test were used to assess OS and DFS between the high- and low-PS_HCC_ groups.

### 2.7. Development and Validation of the Pathomics Nomogram for Prognosis

In the training cohort, univariate Cox regression analyses were performed for OS and DFS, incorporating both PS_HCC_ and clinicopathological features, with variables showing *p* < 0.05 selected for subsequent multivariable analysis. Backward stepwise Cox regression identified the independent predictors. Based on these predictors, two pathomics nomograms were constructed to estimate OS and DFS.

To assess the discrimination of the pathomics nomograms, area under the receiver operating characteristic curve (AUROC) and concordance index (C-index) were calculated. Calibration curves were generated to evaluate the alignment between predicted and actual survival probabilities, and decision curve analysis (DCA) was applied to assess the nomograms’ clinical utility. These nomograms were further validated in the external cohort to confirm their discrimination, calibration, and clinical applicability.

### 2.8. Incremental Value of the PS_HCC_ for Prognosis Prediction

The incremental prognostic value of the PS_HCC_, when added to clinicopathological models including clinicopathological characteristics only, was evaluated using C-index and AUROC. Comparisons of C-indexes and AUROCs at 5 years between models were conducted using the z-score and DeLong tests.

### 2.9. Statistical Analysis

Continuous variables were analyzed using the t-test, while categorical variables were examined using the χ^2^ test. The Kaplan–Meier method, along with the log-rank test, was employed to assess OS and DFS. Univariate and multivariate Cox regression analyses were performed, with HR and 95% CI calculated for each variable. All statistical analyses were carried out using R (version 4.1.3) software. All *p*-values were two-sided, with statistical significance set at *p* < 0.05.

## 3. Results

### 3.1. Patient Characteristics

The clinicopathologic characteristics of patients in the training (n = 268) and validation (n = 121) cohorts are detailed in Table 1. Among the 389 participants, 343 (87.3%) were male. No significant differences in clinicopathological features were observed between the training and validation cohorts. In the training cohort, the median follow-up duration [interquartile range (IQR)] was 57 months (26–79), with 5-year OS and DFS rates of 51.6% and 44.2%, respectively (Figure S1A,B). The validation cohort had a median (IQR) follow-up of 66 months (33–80), with corresponding 5-year OS and DFS rates of 60.9% and 46.7% (Figure S1C,D).

### 3.2. Construction of the PS_HCC_

**Figure 2:**
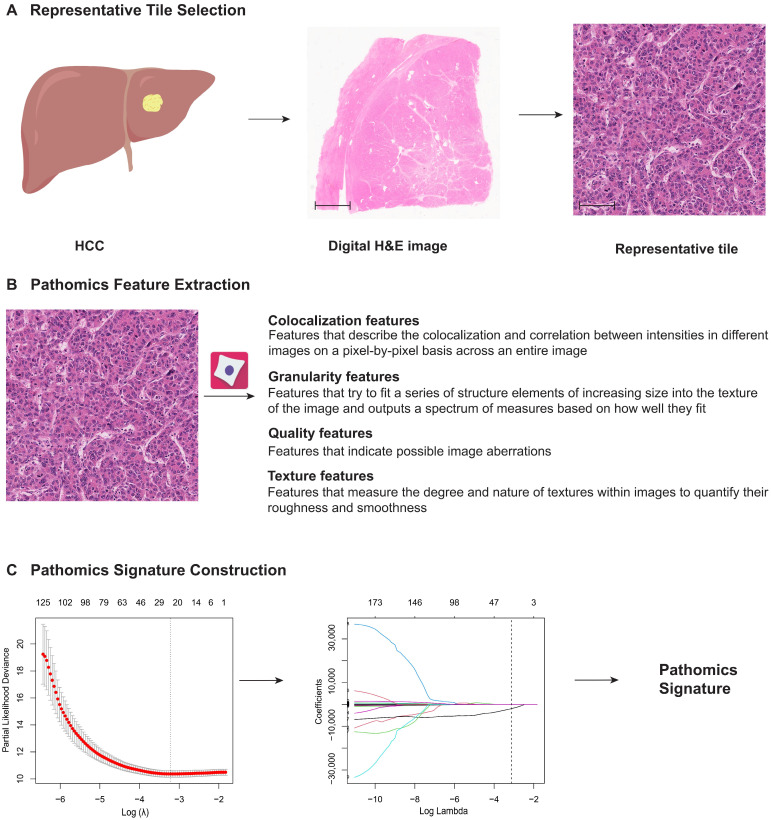
Construction framework of the PS_HCC_. (**A**) Ten regions of interest (ROI), each with a field of view of 1000 × 1000 μm and containing the highest density of tumor cells, were randomly selected from the digital H&E-stained images for analysis. Scale bars: 3000 μm and 50 μm. (**B**) A total of 351 pathomics features were extracted from these representative H&E tiles. (**C**) The PS_HCC_ was constructed using a least absolute shrinkage and selection operator Cox regression model in the training cohort. PS_HCC_: pathomics signature of hepatocellular carcinoma; H&E: hematoxylin and eosin.

The PS_HCC_ construction framework is depicted in Figure 2. In the training cohort, a 24-feature-based PS_HCC_ was developed using the LASSO Cox regression model (Figure S2). The formula used to compute the PS_HCC_ is provided in the Supplementary Information, from which the PS_HCC_ of the validation cohort was directly derived.

### 3.3. Association of the PS_HCC_ with Prognosis

**Figure 3:**
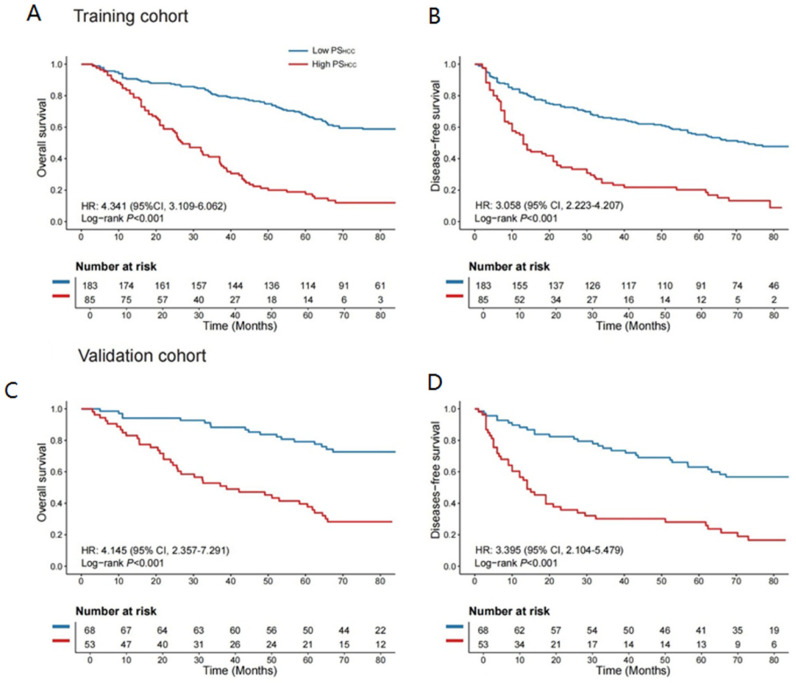
Kaplan–Meier survival curves based on PS_HCC_ levels. (**A**,**B**) Kaplan–Meier curves depicting differences in OS and DFS between high- and low-PS_HCC_ groups in the training cohort. (**C**,**D**) Kaplan–Meier curves showing OS and DFS differences between high- and low-PS_HCC_ groups in the validation cohort. Left panel: OS; right panel: DFS. OS and DFS comparisons between the two groups were performed using a two-sided log-rank test. OS: overall survival; DFS: disease-free survival; PS_HCC_: pathomics signature of hepatocellular carcinoma.

The optimal PS_HCC_ cutoff value, determined as 14.469 via the “survminer” package in the training cohort (Figure S3), was used to categorize all patients into high- and low-PS_HCC_ groups. High-PS_HCC_ patients in the training cohort had significantly shorter OS and DFS than their low-PS_HCC_ counterparts, with hazard ratios (HRs) of 4.341 (95% CI: 3.109–6.062; log-rank *p* < 0.001) and 3.058 (95% CI: 2.223–4.207; log-rank *p* < 0.001), respectively (Figure 3A).

Similar analyses were performed in the validation cohort, where high-PS_HCC_ patients also exhibited significantly reduced OS and DFS compared to low-PS_HCC_ patients, with HRs of 4.145 (95% CI: 2.357–7.291; log-rank *p* < 0.001) and 3.395 (95% CI: 2.104–5.479; log-rank *p* < 0.001), respectively (Figure 3B). The PS_HCC_ demonstrated robust performance in estimating 2-, 3-, and 5-year OS and DFS across both cohorts (Figure S4). Furthermore, it remained an independent prognostic factor when stratified by clinical and pathological variables, underscoring its prognostic value (Figures S5–S8).

### 3.4. Construction and Validation of the Pathomics Nomograms

**Figure 4:**
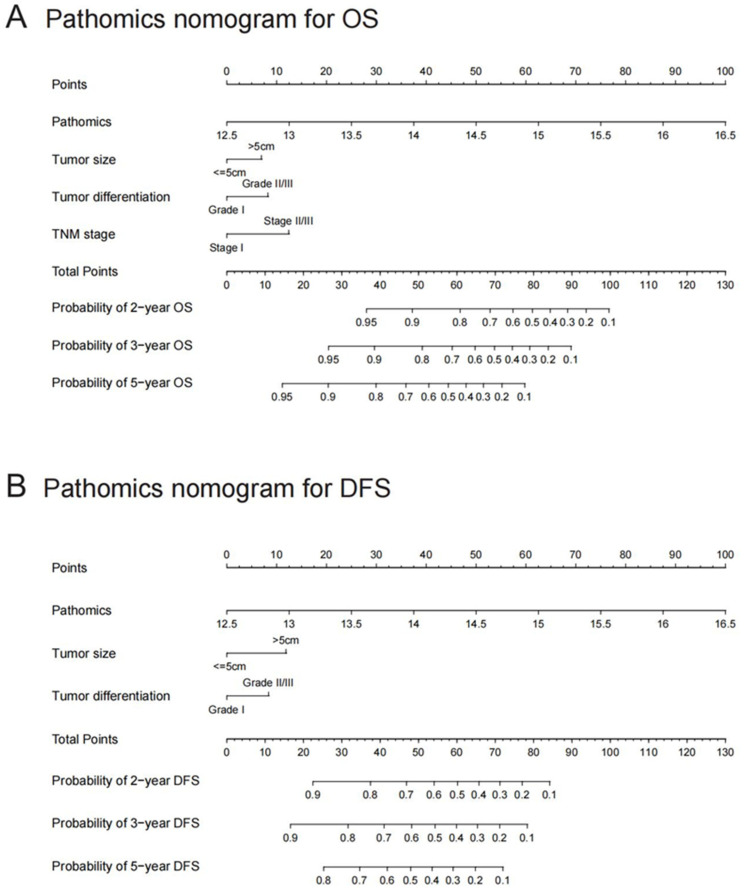
Pathomics nomograms for predicting OS and DFS. (**A**) Pathomics nomogram for overall survival (OS). (**B**) Pathomics nomogram for disease-free survival (DFS). The patient’s position on the PS_HCC_ axis is first identified, followed by drawing a line straight upward to the Points axis to determine the corresponding score. This process is repeated for each variable, and the points from all factors are summed. The total score is then located on the Total Points axis, and a line is drawn straight down to determine the patient’s survival probability. OS: overall survival; DFS: disease-free survival; PS_HCC_: pathomics signature of hepatocellular carcinoma.

In the univariate Cox regression analysis, PS_HCC_, AFP, tumor size, tumor number, differentiation grade, vascular invasion, and TNM stage were significantly linked to OS, while PS_HCC_, AFP, tumor size, tumor number, vascular invasion, and TNM stage were significantly associated with DFS (Table 2). Multivariate Cox regression further identified PS_HCC_, tumor size, tumor number, and differentiation grade as independent predictors for OS, with PS_HCC_, tumor size, and tumor differentiation being independent risk factors for DFS. Consequently, two pathomics nomograms were developed by incorporating these independent factors to predict OS and DFS (Figure 4).

In the training cohort, the pathomics nomogram achieved a concordance index (C-index) of 0.761 (95% CI, 0.727–0.795) for OS and 0.703 (95% CI, 0.667–0.739) for DFS. Time-dependent receiver operating characteristic (ROC) curves showed area under the curve (AUROC) values of 0.827 (95% CI, 0.771–0.883), 0.823 (95% CI, 0.770–0.877), and 0.852 (95% CI, 0.807–0.898) at 2, 3, and 5 years for OS, and 0.791 (95% CI, 0.735–0.846), 0.799 (95% CI, 0.747–0.852), and 0.777 (95% CI, 0.720–0.834) for DFS at the same intervals (Figure S9A,B).

In the validation cohort, the pathomics nomogram produced a C-index of 0.774 (95% CI, 0.714–0.834) for OS and 0.720 (95% CI, 0.668–0.772) for DFS. The AUCs for OS were 0.830 (95% CI, 0.740–0.919), 0.841 (95% CI, 0.757–0.928), and 0.813 (95% CI, 0.731–0.895) at 2, 3, and 5 years, while DFS AUCs were 0.784 (95% CI, 0.702–0.866), 0.797 (95% CI, 0.71–0.876), and 0.752 (95% CI, 0.664–0.840) at corresponding time points (Figure S9C,D).

Calibration curves showed strong concordance between the predicted and actual survival rates at 2, 3, and 5 years in both cohorts (Figure S10). Decision curve analysis further confirmed that pathomics nomograms provided greater net benefit across a wider range of reasonable threshold probabilities compared to clinicopathological models (Figure S11).

### 3.5. Comparison Between the Pathomics Nomograms and Traditional Models

To investigate the incremental value of the PS_HCC_ added to traditional models for predicting prognosis, we constructed two traditional models including tumor size, tumor number, and differentiation grade to estimate OS and DFS. In the training cohort, the traditional models achieved a C-index of 0.681 (95% CI, 0.641–0.721) for OS and 0.615 (95% CI, 0.573–0.657) for DFS. Pathomics nomograms, integrating PS_HCC_ with clinicopathological risk factors, significantly improved the C-index to 0.761 (95% CI, 0.727–0.795, *p* < 0.001) for OS and 0.703 (95% CI, 0.667–0.739, *p* < 0.001) for DFS. These results could also be found in the validation cohort (Table S2). Similarly, the AUROCs of the traditional models were 0.734 (95% CI, 0.673–0.795) for OS and 0.654 (95% CI, 0.591–0.718) for DFS at 5 years. In contrast, the pathomics nomograms yielded significantly higher AUROCs of 0.852 (95% CI, 0.807–0.898, *p* < 0.001) for OS and 0.777 (95% CI, 0.720–0.834, *p* < 0.001) for DFS at 5 years (Figure S12), and these results could also be found in the validation cohort.

## 4. Discussion

In this study, the PS_HCC_ was established through digital H&E images and LASSO Cox regression, demonstrating a significant association with prognosis in HCC individuals with cirrhosis. Additionally, two pathomics nomograms were developed by combining the PS_HCC_ with clinicopathological predictors to assess OS and DFS. These newly constructed nomograms exhibited superior discrimination and calibration, outperforming traditional models based solely on clinicopathological factors. Validation cohort results reinforced the generalizability of the PS_HCC_, indicating its potential utility as a prognostic tool for individualized HCC management.

Accurate prognosis prediction is vital for effective risk stratification and improving survival in HCC, a highly heterogeneous disease often complicated by cirrhosis. Characterized by extracellular matrix remodeling and an immunosuppressive microenvironment, cirrhotic HCC presents challenges for conventional staging systems, which fail to guide precision treatment effectively. Pathomics, by systematically extracting histopathological features, holds promise for enhancing prognosis prediction across various cancers. This study is the first to utilize a pathomics signature to predict HCC prognosis within a cirrhotic liver.

Leveraging image-processing software and machine learning, an accessible and reproducible method was developed for clinicians to incorporate pathomics signatures into routine clinical workflows. CellProfiler, a free, open-source tool, was employed to automatically extract quantitative, high-dimensional pathomics features from H&E-stained images, proving effective in digital histopathology [24]. LASSO Cox, a widely used machine learning technique, identified prognostically relevant features by adjusting feature coefficients based on the lambda penalty parameter. This model was applied for dimensionality reduction, resulting in the construction of a clinically applicable pathomics classifier.

Significant efforts have been made to identify complementary prognostic biomarkers in HCC. In our previous studies, we developed a stemness-based classifier and a 6-gene signature for prognosis prediction. Gao et al. introduced a 9-immune-related lncRNA model to predict OS in HCC [23,25,26]. However, the high cost has significantly impeded the translation of these methods into clinical practice. While radiomics analyses have demonstrated favorable predictive value for HCC, the complexity of medical image processing and the lack of sufficient validation hinder their clinical application [27,28,29]. Pathomics, however, offers the potential to be integrated with other omics approaches to enhance risk stratification in various cancers. For instance, Wang et al. constructed a nomogram by incorporating pathomics, radiomics, and Immunoscore to predict lung metastasis in colorectal cancer [30]. Similarly, a multi-classifier system combining genomics and pathomics has been proposed to predict recurrence in papillary renal cell carcinoma [31]. It is anticipated that future multi-omics analyses, including genomic data, radiomics, immune infiltration status, and pathomics, will refine risk stratification and prognosis prediction for HCC.

Despite numerous studies presenting pathomics analyses of digital H&E-stained images in HCC, few have been translated into clinical practice [32,33]. One primary obstacle is that most reported methods rely on deep neural networks, with inaccessible codes, limiting the ability of researchers to develop or test deep learning-based models on their own data [34,35]. In contrast, open-source software like CellProfiler provides an accessible and user-friendly platform for pathomics analysis. Additionally, deep neural network-derived pathomics features often suffer from the “black box” issue, as they are not easily interpretable [32,33]. In contrast, the features extracted using CellProfiler are well-defined and interpretable, offering supplementary insights into cellular and histological characteristics [36,37].

Liver reserve function is a prognostic factor for HCC. Hepatectomy is recommended to resectable patients with Child–Pugh Class A and highly selected patients with Child–Pugh Class B liver function according to National Comprehensive Cancer Network (NCCN) Guidelines for Hepatocellular Carcinoma [38]. Thus, most patients in this study were Child–Pugh A. The albumin–bilirubin (ALBI) grade, calculated based on only albumin and total bilirubin, is also known to be predictive of prognosis for patients with HCC [39,40]. In univariate and multivariate analysis, we found that the Child–Pugh grade was not associated with the survival, which may be attributed to the majority of patients enrolled in this study having well-preserved liver function (Child–Pugh A). Importantly, PS_HCC_ remained an independent predictor for OS and DFS.

To offer a practical tool that aids clinicians in decision-making for HCC with cirrhosis, two pathomics nomograms were developed by integrating PS_HCC_ with clinicopathological risk factors. These nomograms exhibited superior prognostic performance compared to traditional clinicopathological models. In clinical practice, patients predicted to have poor prognosis could receive more tailored treatment and intensive monitoring to reduce the risk of recurrence. The pathomics nomograms we constructed outperformed prognostic models based on clinicopathological factors [41,42,43], with higher C-index and AUROC. A number of studies integrating pathomics signature and clinicopathological factors showed satisfactory performance in predicting the prognosis of HCC [44,45], yet their generalizability was limited due to the single-center designs or small sample sizes.

The two pathomics nomograms are well-suited for clinical application. First, tumor size and number, routinely assessed in clinical practice, are easily accessible post-hepatectomy. Second, the pathomics signature, derived from standard H&E-stained images, is straightforward to compute using the proposed workflow. Importantly, this process imposes no additional financial burden on patients, emphasizing the PS_HCC_’s translational value.

This study has certain limitations. As a retrospective study, it is subject to inherent biases despite external validation efforts to enhance reliability. Additionally, the study draws from two medical institutions in China, with the majority of patients having hepatitis B virus-related cirrhosis. Prospective randomized trials across diverse populations, including alcohol-related liver disease and nonalcoholic fatty liver disease, are needed to further assess the PS_HCC_’s predictive performance and clinical utility. Finally, we were unable to analyze the association between PS_HCC_ and adjuvant therapy response due to the lack of this information. Thus, further investigations should focus on the relationship between PS_HCC_ and adjuvant therapy response.

## 5. Conclusions

In summary, the PS_HCC_ demonstrated a significant association with prognosis in HCC with cirrhosis. By incorporating the PS_HCC_ with established clinicopathological predictors, two pathomics nomograms were developed and validated, outperforming traditional models in prognostic accuracy. These nomograms offer promising potential for guiding personalized management of HCC in patients with cirrhosis.

## Figures and Tables

**Table 1:** Characteristics of the patients in the training and validation cohorts.

Characteristics	Patients, No. (%)	Patients, No. (%)	*p*
	Training Cohort (n = 268)	Validation Cohort (n = 121)	
**Age, year**			0.282
≤50	111 (41.4)	44 (36.4)	
>50	157 (58.6)	77 (63.6)	
**Sex**			0.406
Female	30 (11.2)	19 (15.7)	
Male	238 (88.8)	102 (84.3)	
**Etiology of liver diseases**			0.546
HBV	234 (87.3)	104 (86.0)	
HCV	21 (7.8)	10 (8.3)	
None or other	13 (4.9)	7 (5.7)	
**HBV**			0.836
Absent	34 (12.7)	17 (14.0)	
Present	234 (87.3)	104 (86.0)	
**Child–Pugh grade**			
A	258 (96.3)	115 (95.0)	0.587
B	10 (3.7)	6 (5.0)	
**AFP** ^1^			0.567
Normal	105 (39.2)	44 (36.4)	
Elevated	163 (60.8)	77 (63.6)	
**Tumor size**			0.174
≤5 cm	163 (60.8)	83 (68.6)	
>5 cm	105 (39.2)	38 (31.4)	
**Tumor number**			0.050
Single	216 (80.6)	108 (89.3)	
Multiple	52 (19.4)	13 (10.7)	
**Tumor encapsulation**			0.563
Yes	154 (57.5)	65 (53.7)	
No	114 (42.5)	56 (46.3)	
**Vascular invasion**			0.266
No	162 (60.4)	81 (66.9)	
Yes	106 (39.6)	40 (33.1)	
**Tumor differentiation**			
Grage I	193 (72.0)	92 (76.0)	0.481
Grade II-III	75 (28.0)	29 (24.0)	
**TNM stage**			0.053
Stage I	134 (50.0)	74 (61.2)	
Stage II-III	134 (50.0)	47 (38.8)	

Categorical variables are compared using a two-sided χ^2^ test. Abbreviations: AFP, Alpha-fetoprotein; TNM, tumor-node-metastasis. ^1^ For AFP, elevated indicates 13.4 μg/L or greater.

**Table 2:** Univariate and multivariate Cox regression analyses of the PS_HCC_ and clinicopathological characteristics for overall survival and disease-free survival in training cohort.

Variables	Univariate Analysis	*p*	Multivariable Analysis	*p*
	HR (95%CI)		HR (95%CI)	
***Overall survival***				
**Age**	0.897 (0.647–1.244)	0.516		
Sex (Female vs. Male)	0.987 (0.587–1.658)	0.961		
HBV (Absent vs. Present)	1.227 (0.730–2.063)	0.439		
Child–Pugh grade (A vs. B)	1.267 (0.560–2.87)	0.571		
Preoperative AFP level (Normal vs. Elevated)	1.529 (1.089–2.148)	0.014		
Tumor size (≤ 5 cm vs. > 5 cm)	1.815 (1.314–2.508)	<0.001	1.649 (1.170–2.323)	0.004
Tumor number (Single vs. Multiple)	2.495 (1.743–3.570)	<0.001		
Tumor differentiation (Grade I vs. Grade II–III)	1.972 (1.411–2.756)	<0.001	1.769 (1.250–2.502)	0.001
Tumor encapsulation (Yes vs. No)	1.091 (0.788–1.510)	0.601		
Vascular invasion (No vs. Yes)	1.785 (1.291–2.467)	<0.001		
AJCC TNM stage (Stage I vs. Stage II-III)	2.773 (1.977–3.889)	<0.001	2.292 (1.243–4.228)	0.008
PS_HCC_	5.351 (3.766–7.605)	<0.001	4.368 (3.083–6.188)	<0.001
***Disease-free survival***				
Age	1.017 (0.744–1.389)	0.335		
Sex (Female vs. Male)	1.299 (0.764–1.389)	0.918		
HBV (Absent vs. Present)	1.188 (0.728–1.940)	0.491		
Child–Pugh grade (A vs. B)	1.074 (0.475–2.428)	0.864		
Preoperative AFP level (Normal vs. Elevated)	1.559 (1.129–2.154)	0.007		
Tumor size (≤5 cm vs. >5 cm)	1.775 (1.305–2.416)	<0.001	1.691 (1.216–2.351)	0.002
Tumor number (Single vs. Multiple)	2.299 (1.618–3.268)	<0.001		
Tumor differentiation (Grade I vs. Grade II–III)	1.716 (1.238–2.377)	0.001	1.548 (1.106–2.166)	0.011
Tumor encapsulation (Yes vs. No)	1.224 (0.899–1.664)	0.198		
Vascular invasion (No vs. Yes)	1.667 (1.224–2.268)	0.001		
TNM stage (Stage I vs. Stage II–III)	2.229 (1.627–3.053)	<0.001		
PS_HCC_	3.565 (2.548–4.988)	<0.001	2.926 (2.080–4.117)	<0.001

Association of all variables with prognosis is analyzed using a two-sided Cox proportional hazard regression analysis. Abbreviations: PS_HCC_, pathomics signature of hepatocellular carcinoma; HR, hazard ratio; CI, confidence interval; AFP, Alpha-fetoprotein. For AFP, elevated indicates 13.4 μg/L or greater.

## Data Availability

Data and codes are available from the corresponding authors on reasonable request.

## Supplementary Materials

The following supporting information can be downloaded at https://www.mdpi.com/article/10.3390/cancers17193192/s1, Figure S1: Kaplan–Meier survival analysis of the training and validation cohorts. Figure S2: Feature selection using a LASSO Cox regression model in training cohor; Figure S3: Selection of the optimum cutoff value for the PS_HCC_. Figure S4: Pathomics signature (PS_HCC_) measured by time-dependent ROC curves in the training and validation cohorts. Figure S5: Kaplan–Meier survival analysis of the OS according to the PS_HCC_ level stratified by clinicopathological variables in the training cohort. Figure S6: Kaplan–Meier survival analysis of the DFS according to the PS_HCC_ level stratified by clinicopathological variables in the training cohort. Figure S7: Kaplan–Meier survival analysis of the OS according to the PS_HCC_ level stratified by clinicopathological variables in the validation cohort. Figure S8: Kaplan–Meier survival analysis of the DFS according to the PS_HCC_ level stratified by clinicopathological variables in the validation cohort. Figure S9: Pathomics nomograms measured by time-dependent ROC curves in the training and validation cohorts. Figure S10: The calibration curves of the pathomics nomogram. Figure S11: Decision curve analyses of different models for OS and DFS. Figure S12: Time-dependent ROC curves of different models for OS and DFS. Table S1: Summary of the pathomics features; Table S2: Comparison of the C-indexes between the pathomics nomograms and clinicopathological models. File S1: PS_HCC_ calculation formula.

## Abbreviations

The following abbreviations are used in this manuscript:

HCC	Hepatocellular carcinoma
PS_HCC_	Pathomics signature of HCC
OS	Overall survival
DFS	Disease-free survival
HR	Hazard ratio
CI	Confidential interval
BCLC	Barcelona Clinic Liver Cancer
HKLC	Hong Kong Liver Cancer
AJCC	American Joint Committee on Cancer
H&E	Hematoxylin and eosin
LASSO	Least absolute shrinkage and selection operator
DCA	Decision curve analysis
ROC	Receiver operating characteristic
AUROC	Area under the receiver operating characteristic curve
NCCN	National Comprehensive Cancer Network
ALBI	Albumin–bilirubin
