# Supplementary material for: Use of a Pathomics Signature to Predict the Prognosis of Hepatocellular Carcinoma with Cirrhosis: A Multicentre Retrospective Study
Source: Cancers (Basel). 2025 Sep 30;17(19):3192. doi: 10.3390/cancers17193192 (PMC12523728; doi:10.3390/cancers17193192)
Supplement: Supplementary file 1 [file cancers-17-03192-s001.zip › cancers-3845360-supplementary.pdf]

Supplemental Materials

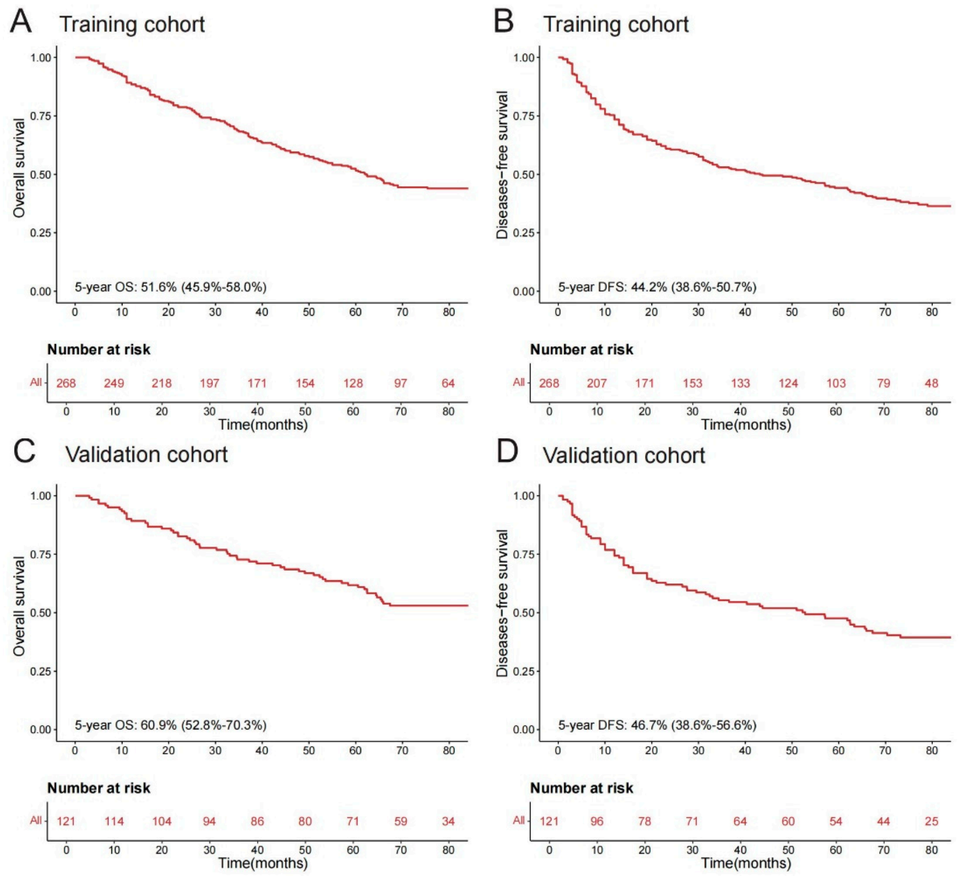

Figure S1: Kaplan–Meier survival analysis of the training and validation cohorts. (A) The OS curve in the training cohort. (B) The DFS curve in the training cohort. (C) The OS curve in the validation cohort. (D) The DFS curve in the validation cohort. OS, overall survival; DFS, disease-free survival.

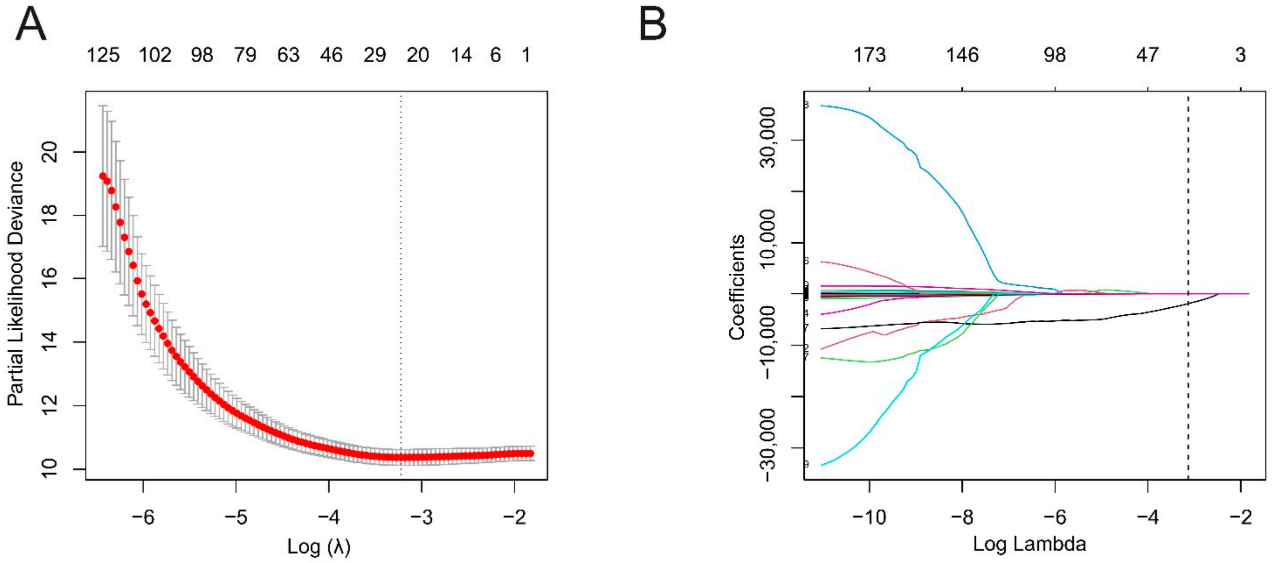

Figure S2: Feature selection using a LASSO-Cox regression model in training cohort. (A) Tuning parameter (λ) selection for the LASSO-Cox regression model via 10-fold cross-validation. The partial likelihood deviance is plotted versus the log (λ) value. Solid vertical lines represent the partial likelihood deviance ± SE. Dotted vertical line is shown at the optimal partial likelihood deviance values, which is calculated by using the minimum criteria. A λ value of 0.03977578, with a log (λ) value of -3.224497, is chosen by 10-fold cross-validation and the minimum criteria. In this cohort, n = 268 patients. (B) Profiles of coefficients from the LASSO-Cox regression model of the extracted pathomics features. A dotted vertical line is shown at the value of log (λ) = -3.224497, at which the optimal λ value results in 24 nonzero coefficients. LASSO, least absolute shrinkage and selection operator.

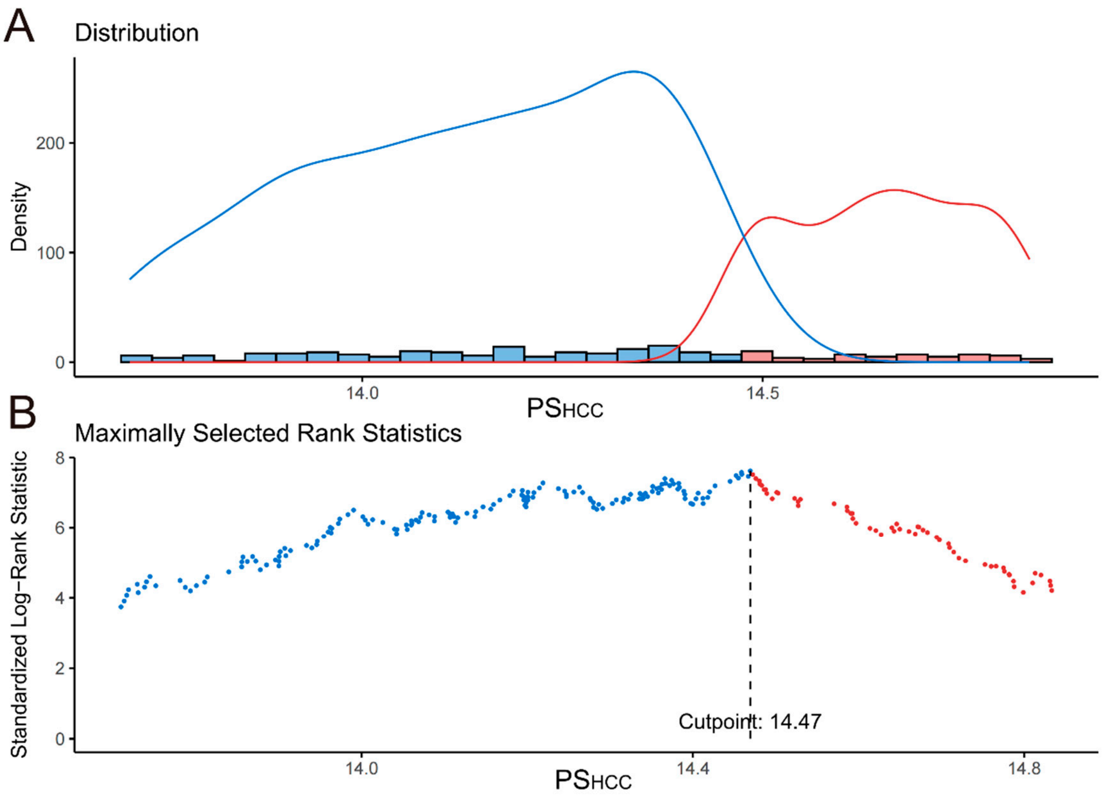

Figure S3. Selection of the optimum cutoff value for the PSHCC. (A) Histogram showing the density distribution for high- and low-PSHCC groups divided by the optimum cutoff value. (B) Scatter plot showing the standardized log-rank statistic value for each PSHCC cutoff value. In this cohort, n = 141 and 123 for patients with high PSHCC and low PSHCC, respectively. The cutoff value is determined by using the two-sided maximally selected rank statistics. PSHCC, pathomics signature of hepatocellular carcinoma.

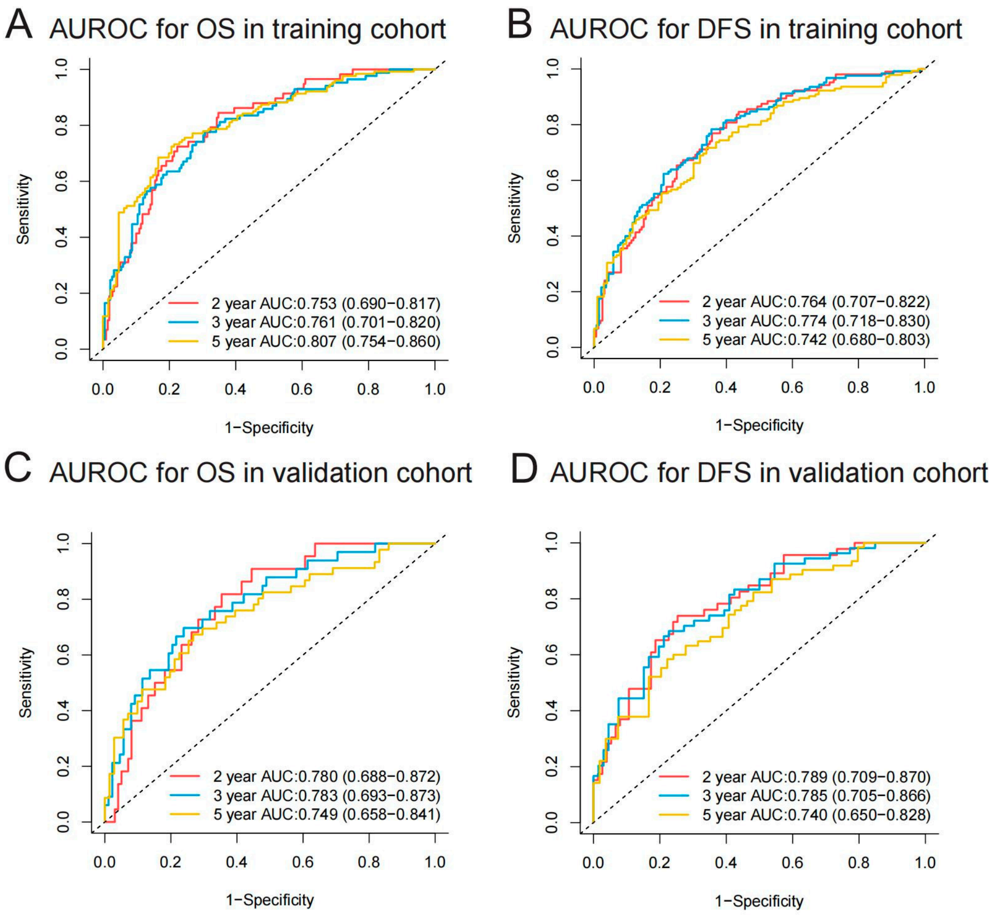

Figure S4. Pathomics signature (PS_{HCC}) measured by time-dependent ROC curves in the training and validation cohorts. (A, B) 2-, 3- and 5-year time-dependent ROC curves of the pathomics signature for DFS and OS prediction in the training cohort. (C, D) 2-, 3- and 5-year time-dependent ROC curves of the pathomics signature for DFS and OS prediction in the validation cohort. Abbreviations: PS_{HCC}, pathomics signature of hepatocellular carcinoma; AUROC, area under the ROC curve; ROC, receiver operating characteristic; DFS, disease-free survival; OS, overall survival; CI, confidence interval.

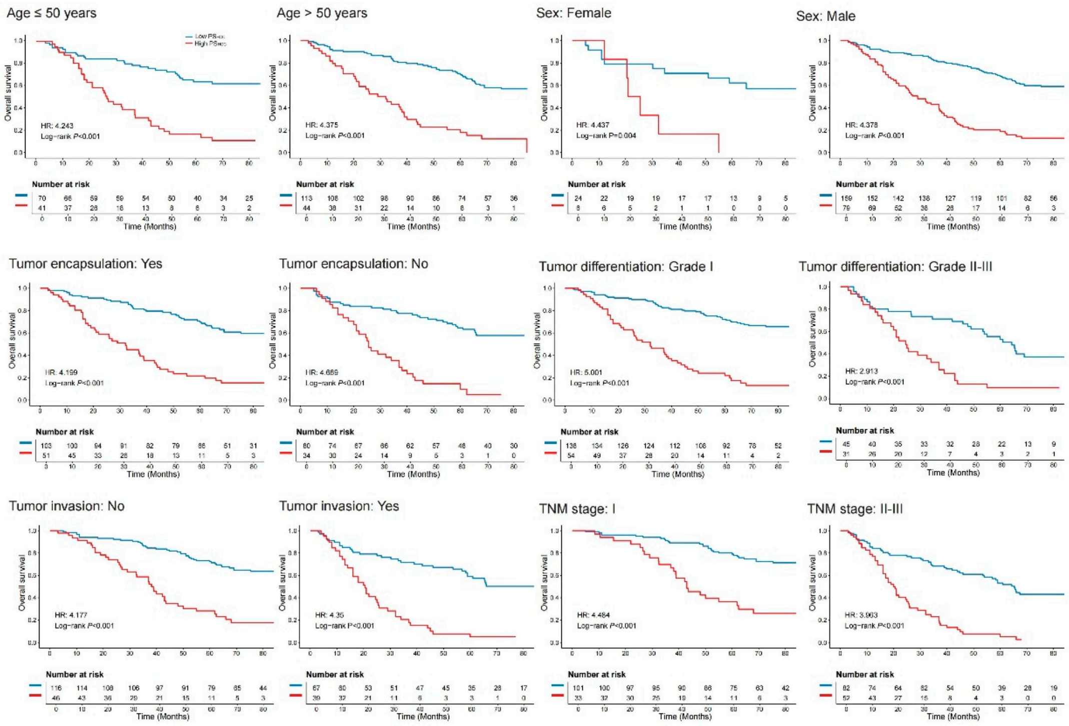

Figure S5. Kaplan–Meier survival analysis of the OS according to the PSHCC level stratified by clinicopathological variables in the training cohort. The comparisons of OS between two groups are performed using a two-sided log-rank test. PSHCC, pathomics signature of hepatocellular carcinoma.; AFP, Alpha-fetoprotein; OS, overall survival.

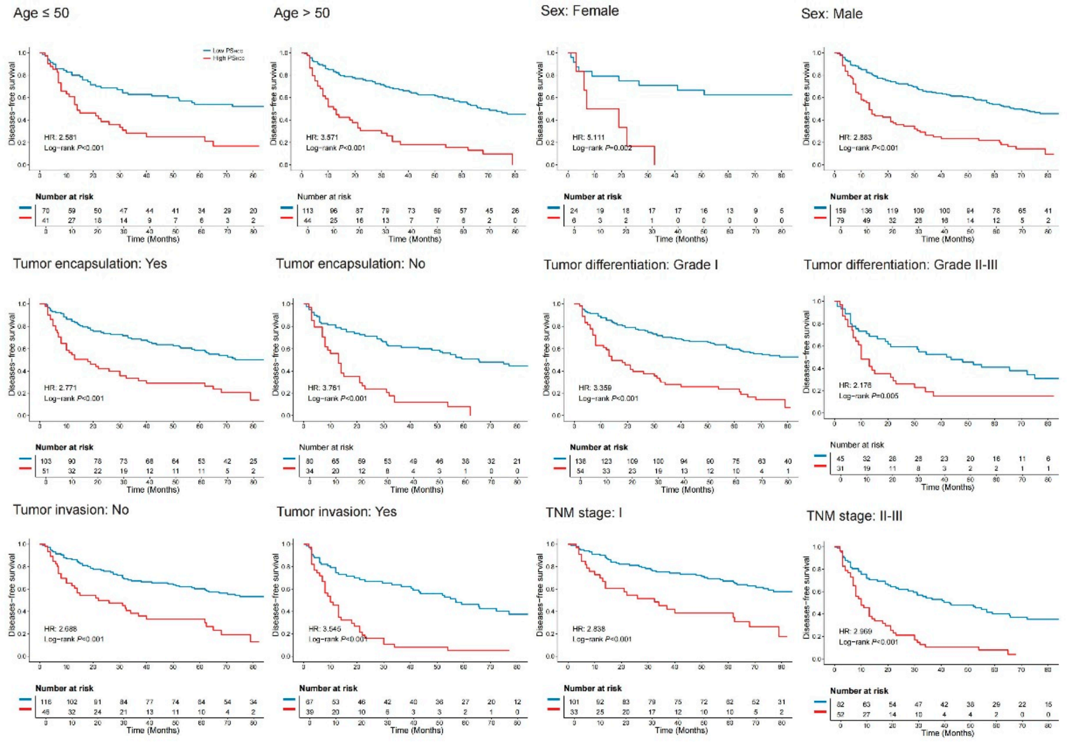

Figure S6. Kaplan–Meier survival analysis of the DFS according to the PSHCC level stratified by clinicopathological variables in the training cohort. The comparisons of DFS between two groups

are performed using a two-sided log-rank test. PSHCC, pathomics signature of hepatocellular carcinoma.; AFP, Alpha-fetoprotein; DFS, diseases-free survival.

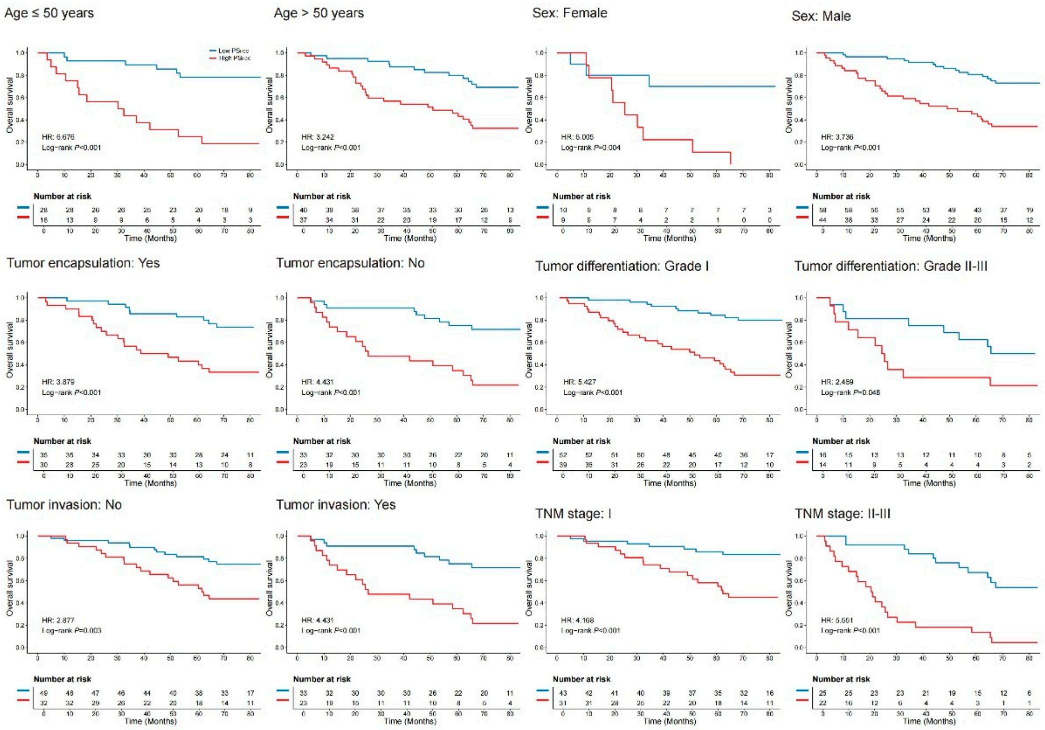

Figure S7. Kaplan–Meier survival analysis of the OS according to the PSHCC level stratified by clinicopathological variables in the validation cohort. The comparisons of OS between two groups are performed using a two-sided log-rank test. PSHCC, pathomics signature of hepatocellular carcinoma.; AFP, Alpha-fetoprotein; OS, overall survival.

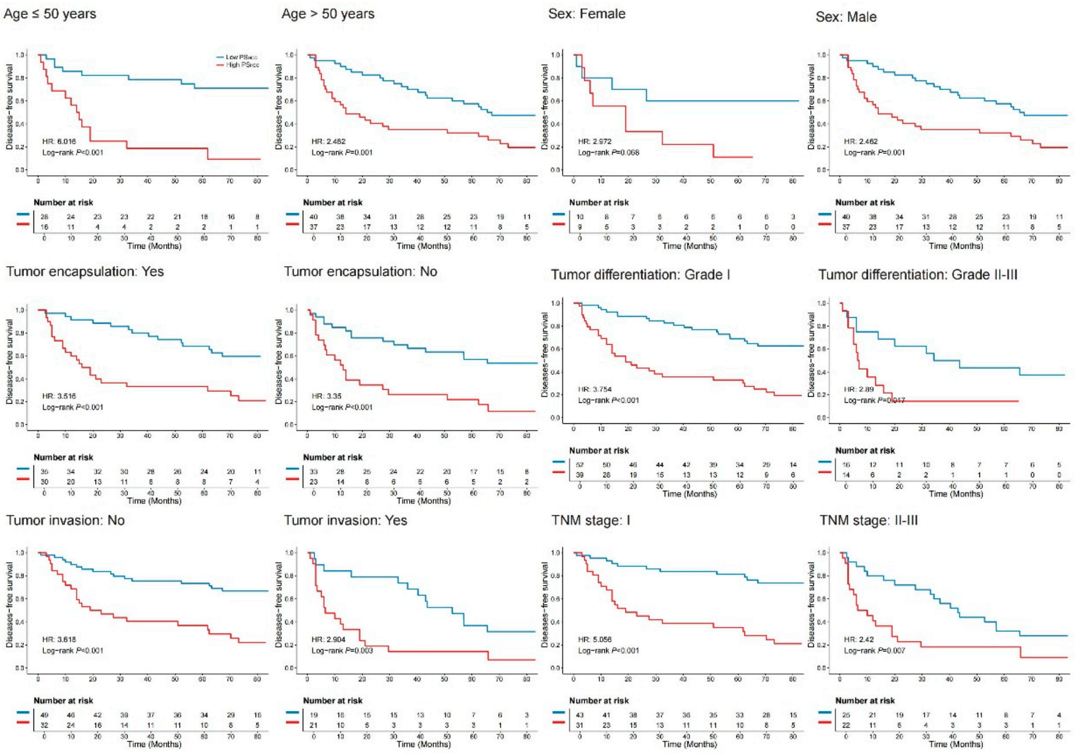

Figure S8. Kaplan–Meier survival analysis of the DFS according to the PSHCC level stratified by clinicopathological variables in the validation cohort. The comparisons of DFS between two groups are performed using a two-sided log-rank test. PS_{HCC}, pathomics signature of hepatocellular carcinoma; AFP, Alpha-fetoprotein; DFS, diseases-free survival.

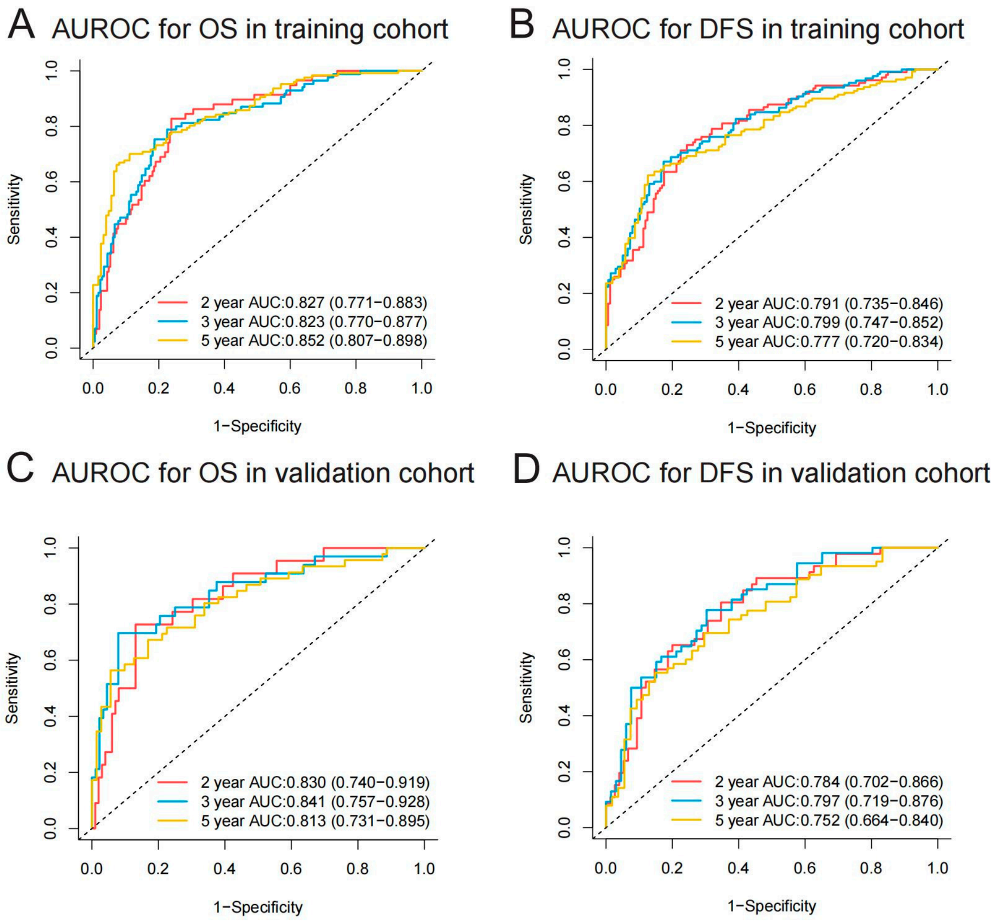

Figure S9. Pathomics nomograms measured by time-dependent ROC curves in the training and validation cohorts. (A, B) 2-, 3- and 5-year time-dependent ROC curves of the pathomics nomograms for DFS and OS prediction in the training cohort. (C, D) 2-, 3- and 5-year time-dependent ROC curves of the pathomics signature for DFS and OS prediction in the validation cohort. Abbreviations: AUROC, area under the ROC curve; ROC, receiver operating characteristic; DFS, disease-free survival; OS, overall survival; CI, confidence interval.

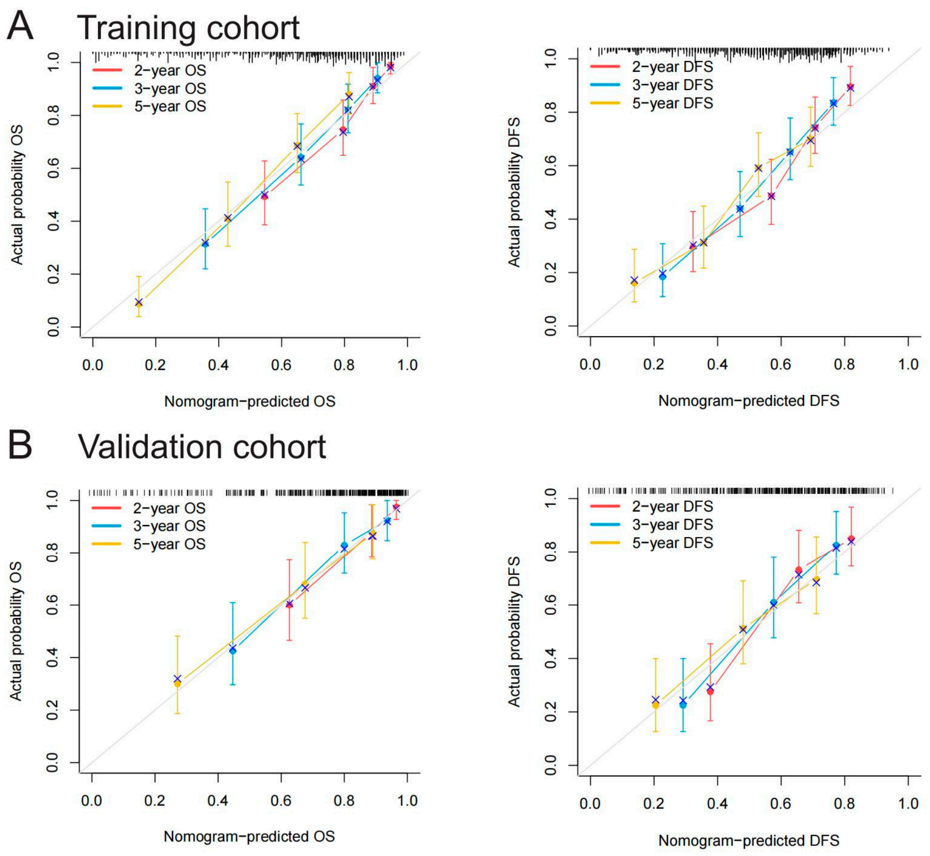

Figure S10: The calibration curves of the pathomics nomogram. (A) Calibration curves of 2-, 3-, and 5-year OS and DFS in the training cohort. (B) Calibration curves of 2-, 3-, and 5- year OS and DFS in the validation cohort. Calibration curves show the calibration of the pathomics nomograms in terms of the agreement between the predicted and actual 2-, 3-, and 5-year outcomes. Left panel: OS; right panel: DFS. Abbreviations: OS, overall survival; DFS, disease-free survival.

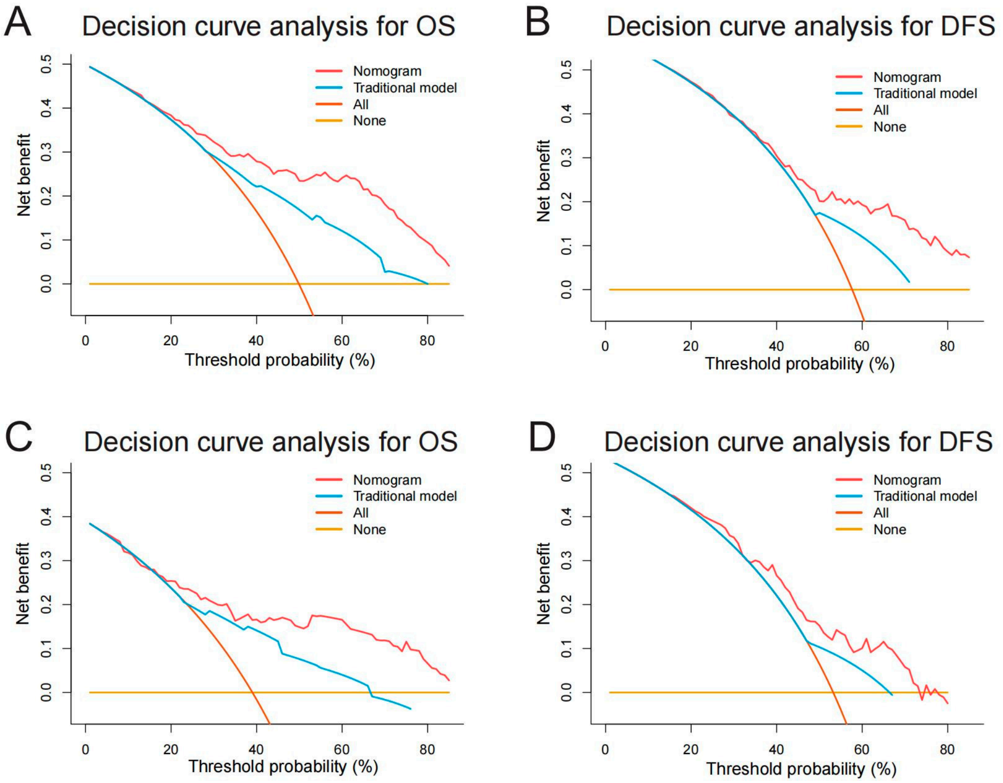

Figure S11. Decision curve analyses of different models for OS and DFS. (A, B) Decision curves of the pathomics nomograms and the clinicopathological models for predicting OS and DFS in the training cohort. (C, D) Decision curves of the pathomics nomograms and the clinicopathological models for predicting OS and DFS in the validation cohort. The *y*-axis represents the net benefit, and the *x*-axis represents the different threshold probabilities. Left panel: OS; right panel: DFS. Abbreviations: OS, overall survival; DFS, disease-free survival.

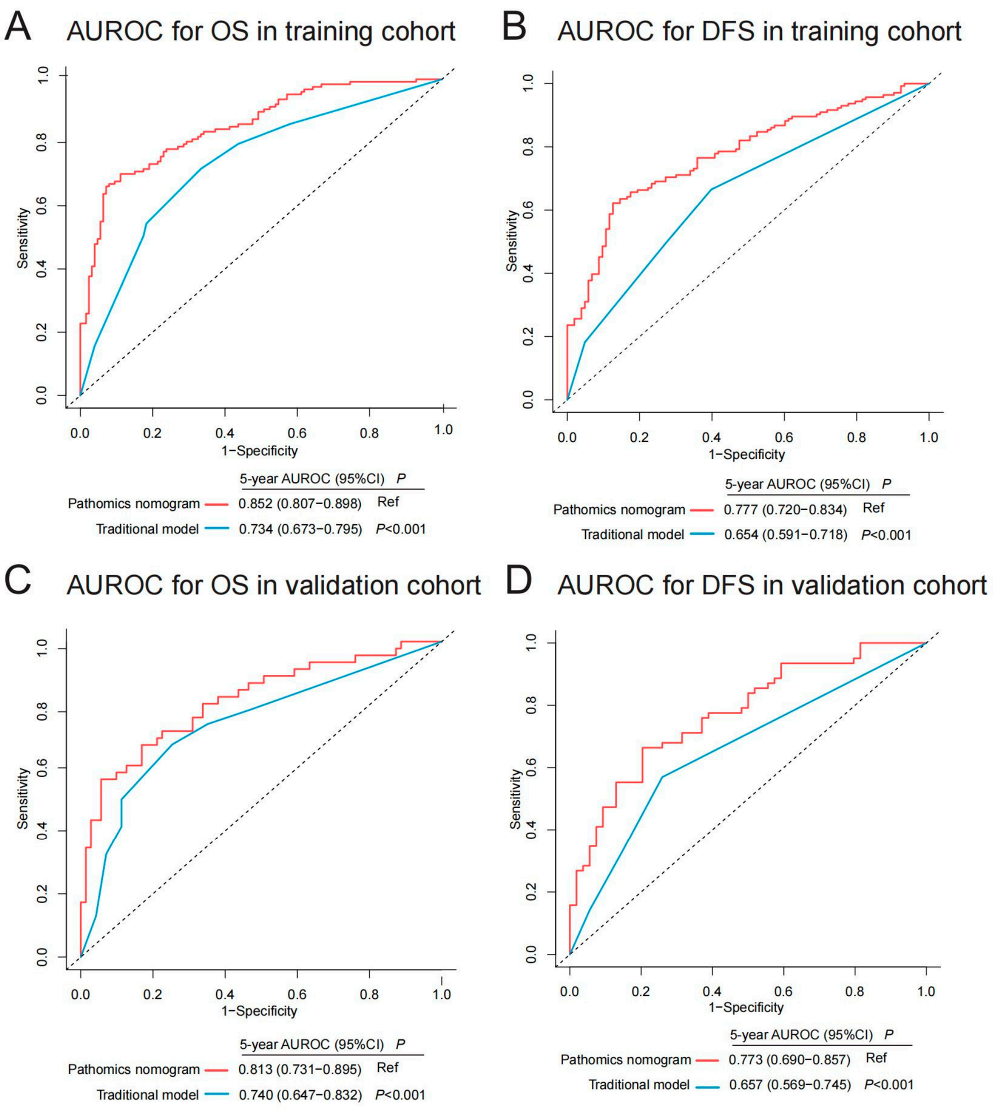

Figure S12. Time-dependent ROC curves of different models for OS and DFS. (A, B) Time-dependent ROC curves of different models for OS and DFS in the training cohort. (C, D) Time-dependent ROC curves of different models for OS and DFS in the validation cohort. Left panel: OS; right panel: DFS. Abbreviations: OS, overall survival; DFS, disease-free survival; ROC, receiver operator characteristic curve; AUROC, area under the ROC curve; CI, confident interval.

Supplemental Tables

Table S1. Summary of the pathomics features.

No.	Feature descriptions
	**Colocalization features**
1	Correlation_Eosin_Hematoxylin
2	Slope_Eosin_Hematoxylin
3	Overlap_Eosin_Hematoxylin
4	Manders_Eosin_Hematoxylin
5	Manders_Hematoxylin_Eosin
6	Costes_Eosin_Hematoxylin
7	Costes_Hematoxylin_Eosin
8	RWC_Eosin_Hematoxylin

9	RWC_Hematoxylin_Eosin
	**Granularity features**
10-57	Granularity_α_β
	**Quality features**
58-60	Correlation_α
61-63	FocusScore_α
64-66	LocalFocusScore_α
67-69	PowerLogLogSlope_α
70-72	MADIntensity_α
73-75	MaxIntensity_α
76-78	MeanIntensity_α
79-81	MedianIntensity_α
82-84	MinIntensity_α
85-87	PercentMaximal_α
88-90	PercentMinimal_α
91-93	Scaling_α
94-96	StdIntensity_α
97-99	ThresholdOtsu_α
100-102	TotalArea_α
103-105	TotalIntensity_α
106-108	Intensity_LowerQuartileIntensity_α
109-111	Intensity_MADIntensity_α
112-114	Intensity_MaxIntensity_α
115-117	Intensity_MeanIntensity_α
118-120	Intensity_MedianIntensity_α
121-123	Intensity_MinIntensity_α
124-126	Intensity_PercentMaximal_α
127-129	Intensity_StdIntensity_α
130-132	Intensity_TotalArea_α
133-135	Intensity_TotalIntensity_α
136-138	Intensity_UpperQuartileIntensity_α
139-141	IntegratedIntensityEdge_α
142-144	IntegratedIntensity_α
145-147	PrimaryObjects_Intensity_LowerQuartileIntensity_α
148-150	PrimaryObjects_Intensity_MADIntensity_α
151-153	PrimaryObjects_Intensity_MassDisplacement_α
154-156	MaxIntensityEdge_α
157-159	PrimaryObjects_Intensity_MaxIntensity_α
160-162	MeanIntensityEdge_α
163-165	PrimaryObjects_Intensity_MeanIntensity_α
166-168	PrimaryObjects_Intensity_MedianIntensity_α
167-171	MinIntensityEdge_α
172-174	PrimaryObjects_Intensity_MinIntensity_α
175-177	StdIntensityEdge_α
178-180	PrimaryObjects_Intensity_StdIntensity_α
181-183	PrimaryObjects_Intensity_UpperQuartileIntensity
184-186	CenterMassIntensity_X_α
187-189	CenterMassIntensity_Y_α
190-192	MaxIntensity_X_α
193-195	MaxIntensity_Y_α
	**Texture features**
196-207	AngularSecondMoment_α_γ
208-219	Contrast_α_γ
220-231	Correlation_α_γ
232-243	DifferenceEntropy_α_γ
244-255	DifferenceVariance_α_γ
256-267	Entropy_α_γ
268-279	InfoMeas1_α_γ
280-291	InfoMeas2_α_γ
292-303	InverseDifferenceMoment_α_γ
304-315	SumAverage_α_γ
316-327	SumEntropy_α_γ

328-339	SumVariance_α_γ
340-351	Variance_α_γ

α represents the type of images, which could be haematoxylin, eosin, and H&E;.

β represents the granular spectrum, which could be 1, 2, 3, 4, …, 15, 16.

γ represents the scale of the texture feature, which could be 0, 1, 2, 3.

MAD, median absolute deviation; RWC, rank weighted colocalization; H&E, hematoxylin and eosin.

Table S2. Comparison of the C-indexes between the pathomics nomograms and clinicopathological models.

Model	Overall survival		Diseases-free survival	
	C-index (95% CI)	*P*	C-index (95% CI)	*P*
**Training cohort**				
Pathomics nomogram	0.761 (0.727-0.795)	Reference	0.703 (0.667-0.739)	Reference
Clinicopathological model	0.681 (0.641-0.721)	<0.001	0.615 (0.573-0.657)	<0.001
**Validation cohort**				
Pathomics nomogram	0.774 (0.714-0.834)	Reference	0.720 (0.668-0.772)	Reference
Clinicopathological model	0.695 (0.626-0.764)	<0.001	0.628 [0.567-0.689]	<0.001

Supplementary File S1. PS_{HCC} calculation formula.

PS_{HCC} = 0.031994975 × Granularity_ H&E_10

-0.021375280 × Granularity_ Eosin _12

+0.129138901 × Granularity_Haematoxylin_14

-0.003034897 × Granularity_Eosin_15

+0.156920823 × Granularity_ Hematoxylin_16

+ 0.146320799 × Granularity_ Eosin _3

-0.028631577 × Granularity_ H&E_ 4

+0.032747107 × Granularity_ H&E_6_

-0.029428052 × Granularity_Eosin_8

+0.089531952 × Granularity_ H&E_8

-0.008273397× Granularity_ Eosin _9

-0.649011472 × LocalFocusScore_Eosin_20

+1.366006591× MaxIntensity_Eosin

+0.301325166 × MedianIntensity_ H&E

+0.002088653 × PercentMinimal_Hematoxylin

-2135.883092829 × PercentMinimal_ H&E

+0.178789431 × MaxIntensity_Eosin

+0.041371640 × MedianIntensity_ H&E

-1.504917663× MassDisplacement_ H&E

+0.511185009 × MaxIntensityEdge_ H&E

-2.784180220× MinIntensityEdge_Hematoxylin

+0.0644249770 × MaxIntensity_X_Eosin

-0.013624828 × MaxIntensity_Y_Hematoxylin

+57.358704962 × AngularSecondMoment_Hematoxylin_3_01_256
